# Retraction: Effects of MicroRNA-146a on the proliferation and apoptosis of human osteochondrocytes by targeting TRAF6 through the NF- κB signalling pathway

**DOI:** 10.1042/BSR-2017-0180C_RET

**Published:** 2021-06-29

**Keywords:** MicroRNA-146a, Osteoarthritis, osteochondrocytes

This Commentary is being retracted from *Bioscience Reports* at the request of the authors. This Commentary has not been retracted for any ethical infringement by its authors, but because significant concerns have been raised on the original article that this commentary refers to (DOI: 10.1042/BSR20160578), which has now been retracted. The Editor-in-Chief and Editorial Board agree with the Retraction.

